# Electrocardiographic Characteristics of Ventricular Arrhythmias Originating from Different Areas Adjacent to the Mitral Annulus

**DOI:** 10.3390/jcdd10080334

**Published:** 2023-08-03

**Authors:** Yi-Fan Lin, Que Xu, Cheng Zheng, Jia-Meng Shao, Bing Shen, Rui-Lin He, Jia-Feng Lin, Yan-Ru Chen

**Affiliations:** 1 Department of Cardiology, The Second Affiliated Hospital and Yuying Children’s Hospital of Wenzhou Medical University, Wenzhou 325000, China; 720125@wzhealth.com (Y.-F.L.); 720128@wzhealth.com (Q.X.); 211325@wzhealth.com (C.Z.); 721041@wzhealth.com (J.-M.S.); 721036@wzhealth.com (B.S.); 720016@wzhealth.com (R.-L.H.); 2 Department of Cardiology, The Third Affiliated Hospital of Wenzhou Medical University and Ruian People’s Hospital, Wenzhou 325000, China

**Keywords:** ventricular arrhythmia, mitral annulus, electrocardiogram, radiofrequency catheter ablation, electrophysiology

## Abstract

Background: This study aimed to explore the electrocardiographic (ECG) characteristics of ventricular arrhythmias (VAs) arising from epicardial and endocardial areas adjacent to the mitral annulus (MA). Methods: This study involved 283 patients with MA-VAs who received radiofrequency catheter ablation at the center. The ECG characteristics of these patients were analyzed retrospectively. Results: The origin of MA-VAs was judged based on the ECG variables. Among all MA-VAs, intrinsicoid deflection time (IDT) > 77 ms or maximum deflection index (MDI) > 0.505 predicted the VAs arising from the epicardium (sensitivity of 70.20% and 73.51%, specificity of 94.70% and 82.58%, positive predictive value (PPV) of 93.81% and 82.84%, and negative predictive value (NPV) of 73.53% and 73.15%). Among all epicardial MA-VAs, the RV1/RV2 ratio > 0.87 predicted the VAs originating from the epicardial anteroseptal wall adjacent to the MA. It had a sensitivity, specificity, PPV, and NPV of 62.86%, 98.06%, 91.67%, and 88.60%, respectively. Among all endocardial MA-VAs, Q(q)R(r) morphology in lead V1 predicted the VAs arising from the endocardial septal wall adjacent to the MA. It had a sensitivity, specificity, PPV, and NPV of 92.98%, 100%, 100%, and 94.94%, respectively. Among all endocardial septal MA-VAs, a predominant positive wave in lead II and a predominant negative wave in lead III predicted the VAs arising from the endocardial midseptal portion adjacent to the MA. It had a sensitivity, specificity, PPV, and NPV of 86.04%, 100%, 100%, and 70.00%, respectively. Conclusion: the ECG characteristics of VAs from the different sites adjacent to the MA can enable judging the arrhythmia’s origin and designing the ablation plan accordingly.

## 1. Introduction

Ventricular arrhythmias (VAs), which include ventricular tachycardia (VT) and premature ventricular contraction (PVC), are one of the most common arrhythmias [1]. As a safe and effective treatment, radiofrequency catheter ablation (RFCA) has undergone rapid progress in the past decade and become a first-line treatment for symptomatic VAs [1,2,3]. Most VAs have an aortic root, left ventricular (LV) septum, or right ventricular outflow tract origin [1,4,5]. However, about 5% to 10% of VAs have a rare origin adjacent to the mitral annulus (MA) [6,7]. MA-VAs can be divided into epicardial MA-VAs and endocardial MA-VAs, which are very different in the ablation treatment [5]. The unique anatomical structure of the MA leads to difficulties for RFCA. Analyzing electrocardiographic (ECG) characteristics as a guidance of RFCA may shorten the ablation time and fluoroscopy time. Previous studies have reported the ECG characteristics of MA-VAs in patients without structural heart disease as the following: right bundle branch block pattern (RBBB), S or s waves in lead V6, and an early precordial transitional zone < V2 [7,8]. After that, many studies focused on ECG characteristics and RFCA of VAs originating from specific areas adjacent to the MA [9,10,11,12,13,14]. However, few have systematically analyzed VA’s ECG characteristics originating from both epicardial and endocardial areas adjacent to the MA. The aim of this study was to explore the ECG characteristics of endocardial and epicardial MA-VAs sites as a useful guide for planning an ablation strategy.

## 2. Materials and Methods

### 2.1. Study Population

This retrospective study analyzed 3435 patients referred for RFCA for VAs to The Second Affiliated Hospital and Yuying Children’s Hospital of Wenzhou Medical University from January 2009 to September 2021. The origin sites were located in areas adjacent to the MA in 283 patients (8.24%). These 283 cases included 132 endocardium cases and 151 epicardium cases. The inclusion and exclusion of patients followed the 2019 HRS/EHRA/APHRS/LAHRS expert consensus statement on the catheter ablation of ventricular arrhythmias [1]. All cases had frequent PVCs/VT (defined as PVCs more than 10,000 counts/24 h) or severe clinical symptoms intolerable to at least two antiarrhythmic drugs (AADs). Prior to ablation, AADs were abandoned for at least five half-lives. Complete physical examination, echocardiography, or coronary angiography (8.83% cases underwent coronary angiography) proved no structural heart diseases in any patient. All patients provided informed written consent before ablation treatment. The hospital’s ethics committee approved this research.

### 2.2. ECG Measurement

This study obtained and analyzed twelve-lead ECGs before RFCA. All ECG measurements were reviewed by three researchers independently, who were blinded to the patients’ ablation outcomes. All ECG measurements were repeated three times for each researcher, the average value of which was used for analysis. ECG analysis focused on the following:

(i) The QRS morphology of the VAs in all 12 leads;
(ii) (The R-wave amplitude measured from the peak of the R-wave to the isoelectric line (mV));
(iii) The R-wave transition zone of VAs: the position of the precordial leads in which the amplitudes of the R and S waves were equal;
(iv) Intrinsicoid deflection time (IDT): the interval measured from the QRS onset to the peak of the R wave in V2 (measure V3 when V2 cannot be measured);
(v) Maximum deflection index (MDI): the ratio of IDT to the maximum QRS duration (the maximum time from the beginning to end of the QRS wave in twelve-lead ECG) [15,16].

### 2.3. Electrophysiological Examination and Radiofrequency Ablation

After stopping antiarrhythmia medication for at least five half-lives, an electrophysiological examination was performed. The catheter (Thermo-cool or Smart Touch, Biosense Webster, Diamond Bar, CA, USA) was guided by a three-dimensional mapping system (Carto3, Biosense Webster, USA) and fluoroscopy. The catheter systemically mapped the right ventricular outflow tract, pulmonary sinus, left ventricular outflow tract, and great cardiac vein. If VAs failed to occur spontaneously, intravenous isoproterenol infusion (2–5 mg/minute) was administered. Those with the following characteristics during the electrophysiological examination were regarded as ideal ablation targets:

(i) The earliest V wave in intracardiac bipolar electrogram was more than 20 ms preceding the QRS onset of VAs;
(ii) The QRS complex waveform of activation mapping was similar to spontaneous VAs in at least 10 leads, or the similarity analyzed by the Carto3 Paso system was greater than 95%.

Radiofrequency energy was delivered once the target site was located. In the epicardial target site, the upper limit impedance, preset power, upper limit temperature, and saline flow rate were preset from 200 Ω to 300 Ω, 25 W to 35 W, 43 °C, and 30 to 60 mL/minute, respectively. In contrast, the saline flow rate was preset to 17 mL/minute in endocardial origin. If the VAs were terminated within 15 s or more and PVCs or non-sustained VTs occurred during ablation at the target site, the additional current was applied for another 60 to 120 s. The target was remapped if the VAs were not terminated after 30 s of ablation. Ablation was considered effective when the VAs was terminated and could not be induced by isoproterenol or programmed stimulation. Each patient underwent ECG monitoring for at least 24 h after RFCA. The treatment achieved acute success when no VAs recurrence within 24 h postoperatively. 

### 2.4. Anatomic Division of Sites Adjacent to MA

**Figure 1:**
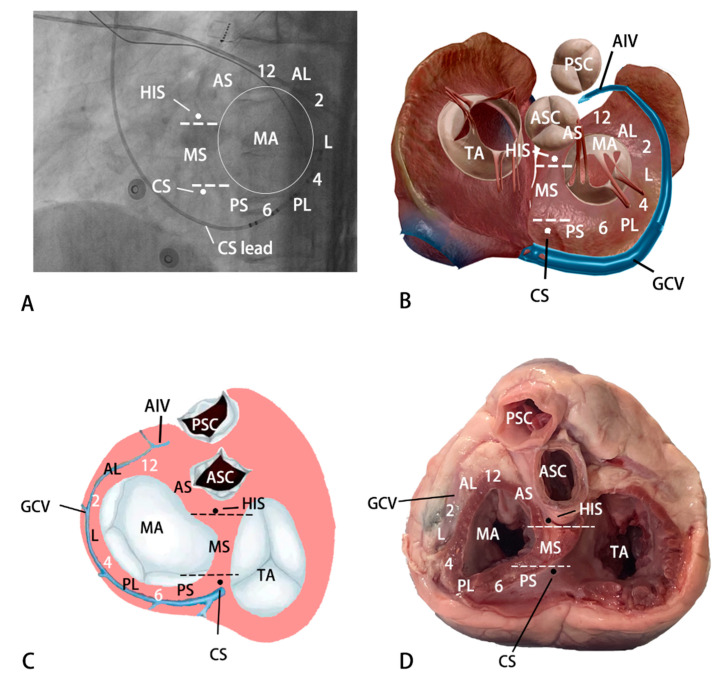
Anatomic division of sites adjacent to MA. PSC, pulmonary sinus cusp; ASC, aortic sinus cusp; GCV, great cardiac vein; TA, tricuspid annulus; MA, mitral annulus; AS, anteroseptal portion; MS, midseptal portion; PS, posteroseptal portion; PL, posterolateral portion; L = lateral portion; AL = anterolateral portion; AIV, anterior interventricular veins. (**A**) The fluoroscopy of sites adjacent to MA. (**B**) Anterior-posterior view of sites adjacent to MA. (**C**,**D**) Posterior-anterior view of sites adjacent to MA.

This study drew a vertical line at the twelve and six o’clock directions of the MA in the left anterior oblique (LAO) view to divide the areas adjacent to MA into the septum and the free wall. This study subdivided it into the following six segments (Figure 1). (i) Anterolateral portion ranging from 12 o’clock (contain) to 2 o’clock; (ii) lateral portion ranging from 2 o’clock (contain) to 4 o’clock; (iii) posterolateral portion ranging from 4 o’clock (contain) to 6 o’clock (contain); (iv) posteroseptal portion ranging from 6 o’clock to the superior margin of the CS; (v) midseptal portion ranging from the superior margin of the coronary sinus ostium (CS) to the inferior margin of His branch; (vi) anteroseptal portion ranging from the inferior margin of His branch to 12 o’clock. In patients with the epicardial origin, the great cardiac and anterior interventricular veins (AIVs) corresponded to the free wall (distal great cardiac vein extending to 12 o’clock) and anteroseptal portion, respectively.

### 2.5. Follow-up and Definition of Outcome

Each patient underwent ECG monitoring for at least 24 h after RFCA. Then, 24 h Holter monitoring was performed 1, 3, 6, and 12 months after RFCA to evaluate the long-term effects. Acute success was defined as the complete elimination of spontaneous or inducible VAs during RFCA and no VA recurrence within 24 h postoperatively. Long-term success was defined as the PVC decreasing by at least 80% 24 h after Holter was performed three months after RFCA compared with that before RFCA. Recurrence was defined as PVC decreasing by less than 80% in three months after RFCA and uncomfortable symptoms reappearing.

### 2.6. Statistical Analysis

The continuous variables were reported as mean ± standard deviations, and categorical variables were expressed as percentages. The two-sample *t*-test compared continuous variables with normal distribution, and Wilcoxon signed-rank tests were used for continuous variables with skewed distribution. The χ^2^ test or Fisher’s exact test compared discontinuous variable groups, and *p* < 0.05 was considered significant. Receiver operating characteristic (ROC) curves obtained the best sensitivity and specificity values. IBM SPSS Statistics (version 26) performed statistical analyses.

## 3. Results

### 3.1. Patient Characteristics

According to the target site location, 283 patients were divided into endocardium and epicardium groups. There were no significant differences in sex, age, the total number of PVCs, and disease duration between the groups. In addition, the proportion of hypertension, diabetes, left ventricular enlargement, types of arrhythmias, left ventricular end-diastolic dimension (LVEDd) and LVEF showed the same trend (Supplementary Table S1). In 36 cases (12.72%), echocardiography showed that the left ventricle was enlarged (the left ventricular end diastolic dimension was 55 mm to 79 mm), and the left ventricular ejection fraction (LVEF) was 36% to 55% (all of them recovered to normal LVEF 0.5 to 1 year after the disappearance of VAs after RFCA). The endocardium group contained 66, 3, 6, 4, 43, and 10 cases from the anterolateral, lateral, posterolateral, anteroseptal, midseptal, and posteroseptal portions, respectively. In contrast, the epicardium group contained 103, 5, 8, and 35 cases from the anterolateral, lateral, posterolateral, and anteroseptal portions, respectively.

### 3.2. Electrophysiological Study and Radiofrequency Catheter Ablation

When the catheter was in a parallel or vertical position compared to the mitral valve annulus, and the catheter tip had a characteristic wobbling motion synchronized with the mitral valve annulus, the catheter tip was located near the MA [17]. Electrophysiological examination showed that the V wave amplitude (>0.5 mV) was greater than the A wave (>0.08 mV). This observation suggested that the target site was near the endocardial MA [10,18]. However, the amplitude of an A wave may be greater than a V wave when the target site is located in the epicardial anterolateral portion. This is because of the large far-field left atrial appendage potential recording. Fluoroscopy and a three-dimensional mapping system further verified that the target was located in the endocardium or epicardium adjacent to the MA.

After RFCA therapy, 255 patients (90.11%) achieved acute success. The success rates of the epicardium and endocardium groups were 85.4% (129/151) and 95.5% (126/132), respectively. The success rates of each subgroup in the epicardium group were 90.3% (93/103, anterolateral), 60.0% (3/5, lateral), 75.0% (6/8, posterolateral), and 77.1% (27/35, anteroseptal). The success rates of each subgroup in the endocardium group were 98.48% (65/66, anterolateral), 100.0% (3/3, lateral), 83.33% (5/6, posterolateral), 50.0% (2/4, anteroseptal), 95.3% (41/43, midseptal), and 100.0% (10/10, posteroseptal). The success rate of RFCA in the endocardium group (96.45%) was significantly higher than that in the epicardium group (85.43%) (*p* < 0.01). The failure rate of patients originating from epicardial anteroseptum (22.86%, 8/35) was higher than that of other epicardial portions (12.07%, 14/116). However, there was no significant difference between them (*p* > 0.05).

**Figure 2:**
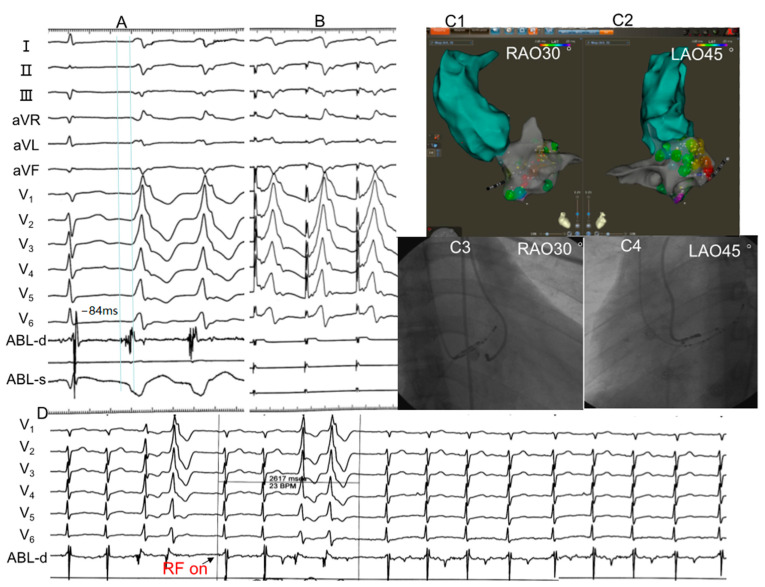
Example of a successful ablation of VAs originating from posterolateral of the endocardial MA. (**A**) Twelve-lead ECG morphology of VAs. RS wave on leads I and aVL, rS wave on leads II, III and aVF, qR wave on lead aVR, R wave on leads V1 to V4, and Rs wave on leads V4 to V6. The local ventricular activation preceded the onset of the QRS complex by −84 ms. (**B**) Small A wave and large V wave can be seen in sinus rhythm, and pacing this site led to a 97.1% concordance with the QRS complex of PVC. (**C1**–**C4**) X-ray images and three-dimensional mapping suggested that the target site located in posterolateral of the endocardial MA. (**D**) RF current was delivered at saline flow rate of 17 mL/min, temperature of 43 °C and power output of 35 W. The VAs were terminated after RF energy delivery for 2617 ms and additional current was applied for another 120 s. After that, PVC could not be induced by intravenous administration of isoproterenol and programmed stimulation. LAO, left anterior oblique; RAO, right anterior oblique; RF on, radiofrequency energy onset.

**Figure 3:**
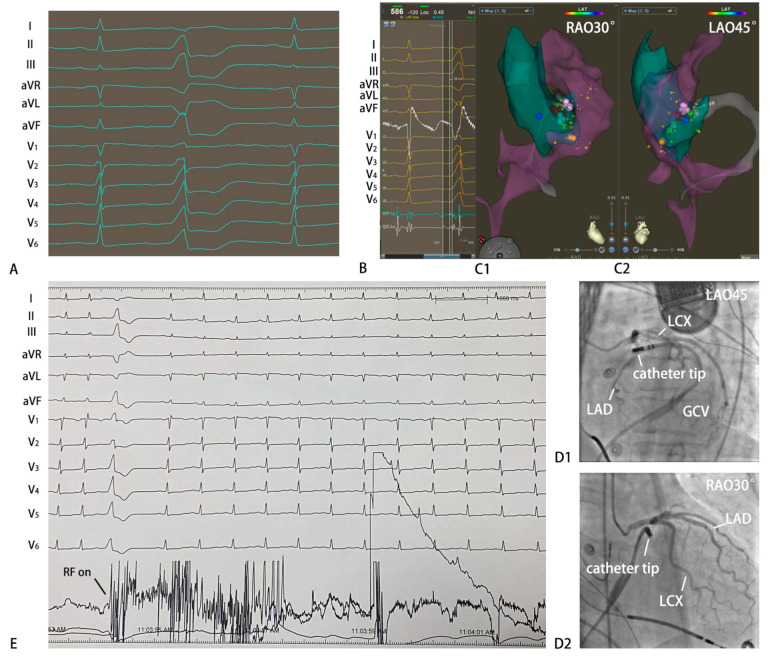
Example of a successful ablation of VAs originating from anterolateral of the epicardial MA. (**A**) Twelve-lead ECG morphology of VAs. QS wave on leads I and aVL, R wave on leads II, III and aVF, QS wave on lead aVR, and Rs wave on leads V1 to V6. (**B**) The local ventricular activation preceded the onset of the QRS complex by −28 ms. (**C1**,**C2**) Three-dimensional mapping suggested that the target site located in anterolateral of the epicardial MA. (**D1**,**D2**) Coronary angiography was performed to clarify the distance between the target and the circumflex branch of coronary artery was more than 5 mm. (**E**) RF current was delivered at saline flow rate of 60 mL/min, temperature of 43 °C, and power output of 30 W. The VAs were terminated and could not be induced after RF energy delivery for 3384 ms and additional current was applied for another 100 s twice. GCV, great cardiac vein; LAD, left anterior descending branch; LCX, left circumflex branch; LAO, left anterior oblique; RAO, right anterior oblique; RF on, radiofrequency energy onset.

The characteristics of the electrophysiological study and RFCA are shown in Supplementary Table S2. The epicardium group had significantly longer procedures (68.0 ± 9.1 vs. 57.4 ± 7.2, *p* < 0.05) and fluoroscopy time (9.8 ± 2.5 vs. 5.3 ± 1.7, *p* < 0.05) than the endocardium group. The complicated anatomy of GCV limited the manipulation of the catheter, so more equipment, such as a Swartz or deflectable sheaths, was needed for support. Presystolic long-duration multicomponent fractionated potential in bipolar mapping was more common in the epicardium group. Typical cases of RFCA of MA-VAs are shown in Figure 2 and Figure 3.

### 3.3. Complications and Follow-up 

The incidence of complications in the epicardium group was significantly higher than that in the endocardium group (*p* < 0.05) (Supplementary Table S3). In the epicardium group, there were eight untreated coronary vein dissection cases. Two cases developed acute pericardial effusion after coronary vein rupture, and both improved after pericardiocentesis. Three cases underwent acute coronary artery injury; two had 35% and 40% of LAD stenosis, respectively. It improved after intracoronary nitroglycerin injection. Delayed pericardial effusion occurred 22 days after RFCA in one case. It improved after pericardiocentesis and corticosteroid therapy. The patients in the endocardium group were not related to the above-mentioned coronary artery and coronary vein injury, but four cases suffered steam pops.

In the epicardium group, 137 cases (90.73%) achieved ablation at the target site, and 129 (85.43%) achieved acute success. In 1 of 22 failure cases, VAs disappeared after follow-up for 1.5 years. In 15 failure cases, VAs still existed. The follow-up was lost in the remaining six failure cases. Recurrence occurred in three cases (2.33%) during follow-up (occurring within three months after RFCA). One of these cases underwent RFCA again and achieved success. Another two patients refused further ablation. Three cases with coronary artery injury or spasm underwent CT angiography three to six months after RFCA. No severe symptoms or complications were observed, except in one patient with delayed pericardial effusion.

In the endocardium group, 126 cases (95.45%) achieved acute success, and recurrence occurred in 4 cases (3.17%). In three of four recurrence cases, ablations were adopted again and succeeded, while ablation was abandoned in the remaining patient. VAs remained during follow-up in four of six failure cases; the other two cases were lost to follow-up.

### 3.4. Electrocardiographic Characteristics of MA-VAs

**Figure 4:**
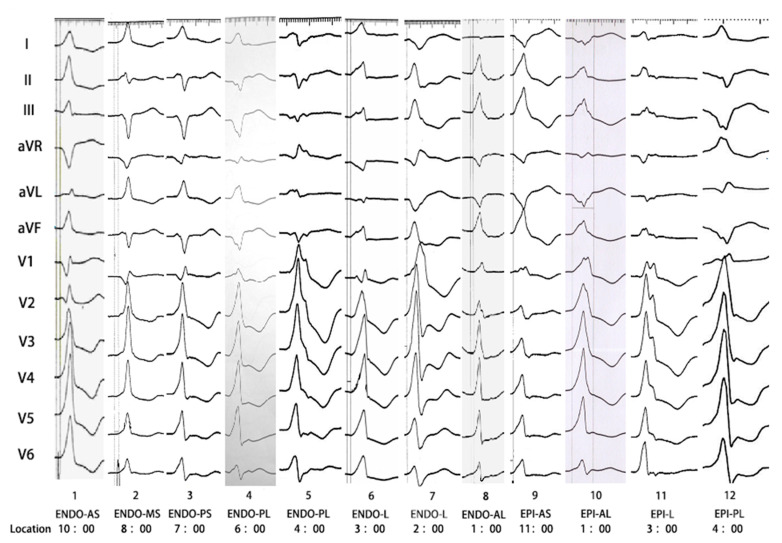
ECG of VAs originating from different areas adjacent to mitral annulus. ENDO = endocardium; EPI = epicardium; AS = anteroseptal; MS = midseptal; PS = posteroseptal; PL = posterolateral; L = lateral; AL = anterolateral.

The electrocardiographic characteristics of VAs originating from the areas adjacent to MA are as follows (Supplementary Table S4 and Figure 4).

(i) The R-wave transition zone of VAs showed a counter-clockwise rotation, was primarily located before lead V1, and positive waves were dominant in leads V2–V6. However, the transitional zone of 19 cases with VAs from epicardial anteroseptal was located in V2–V3.
(ii) VAs originating from the anterior part adjacent to the MA (anterolateral or anteroseptal) usually showed positive waves in the inferior leads (100%), while a posterior origin (posteroseptal or posterolateral) often showed negative waves in the inferior leads (95.83%). In addition, 86.04% (37/43) patients with VAs from the midseptum showed a predominant positive wave in lead II and a predominant negative wave in lead III.
(iii) In the endocardium group, VAs arising from the septal or posterolateral portion often showed a predominant positive R wave in lead I (98.41%), while the anterolateral and lateral portions showed a predominant negative R wave (98.55%). However, a predominant positive wave in lead I was rare in the epicardial anteroseptum group. Among 35 epicardial anteroseptum cases, 24 cases of rs wave, 2 cases of qr wave, and 9 cases of qs wave were observed in lead I. Furthermore, qs wave only accounted for 40.78% (42/103) in epicardial anterolateral group.
(iv) The notch in the upstroke of the R wave was more often observed in the epicardium (91.39%) than in the endocardium (24.24%). In contrast, the notch in the downstroke of the R wave was more often observed in the lateral portion (74.86%) than in the septum (36.96%).

### 3.5. R_V1_/R_V2_ Ratio Diagnosed VAs Originating from the Epicardial Anterolateral Portion Adjacent to MA

**Figure 5:**
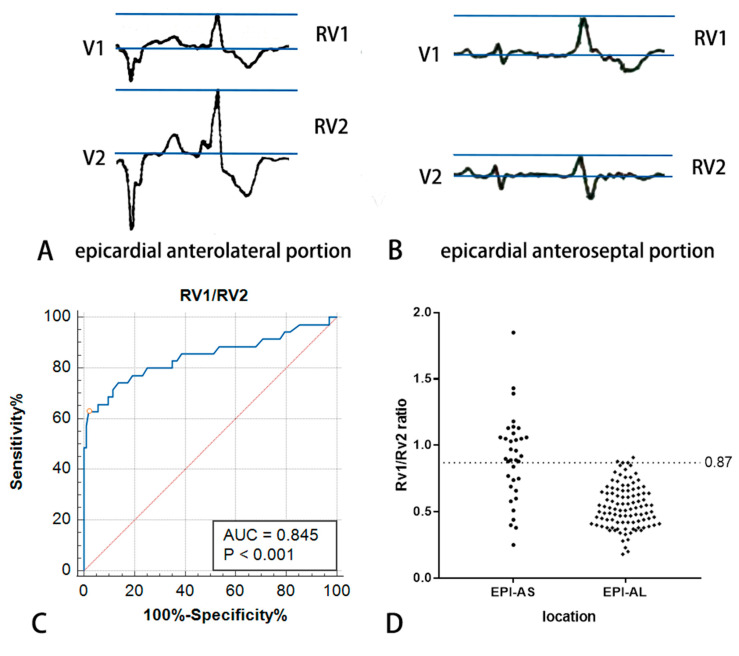
The measurement of R_V1_/R_V2_ ratio and ROC curve for diagnosing VAs originating from epicardial anterolateral portion adjacent to MA. (**A**) ECG of a case with VA arising from epicardial anterolateral portion adjacent to mitral annulus. R_V1_= 0.62 mV, R_V2_ = 1.15 mV and R_V1_/R_V2_ ratio = 0.54. (**B**) ECG of a case with VA arising from epicardial anteroseptal portion adjacent to mitral annulus. Abrupt loss of R wave in lead V2 could be observed. R_V1_ = 0.46 mV, R_V2_ = 0.25 mV and R_V1_/R_V2_ ratio = 1.84. (**C**,**D**) R_V1_/R_V2_ ratio for differentiation of EPI-AS and EPI-AL VAs (ROC curves and scatter plots). Abbreviation: EPI-AS, epicardial anteroseptal portion; EPI-AL, epicardial anterolateral portion; AUC, Area Under Curve. Blue line is ROC curve for diagnosing VAs originating from epicardial anterolateral portion adjacent to MA. Red line is pure opportunity line. Black dots indicate cases in EPI-AS and EPI-AL.

An abrupt loss of the R wave was observed in lead V2 in 16 cases (R_V1_ > R_V2_). All of these cases had an epicardial anterolateral origin. The R_V1_/R_V2_ ratio in VAs originating from the epicardial anteroseptal wall adjacent to MA was significantly higher than other epicardial MA-VAs. The ROC curve shows R_V1_/R_V2_ ≥ 0.87, strongly indicating an epicardial anteroseptal origin (Figure 5).

### 3.6. IDT and MDI Predicted the VAs Arising from the Epicardium

**Figure 6:**
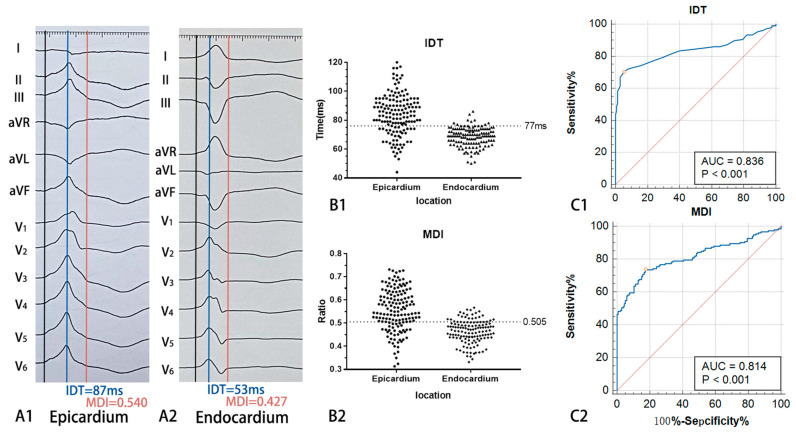
The measurement of IDT and MDI and ROC curve for diagnosing VAs originating from epicardium. (**A1**) ECG of a case in epicardium group. (**A2**) ECG of a case in endocardium group. (**B1**,**B2**) Scatter plots of IDT and MDI in two groups. (**C1**,**C2**) ROC curves for diagnosing VAs originating from epicardium. Abbreviation: IDT, intrinsicoid deflection time; MDI, the maximum deflection index; AUC, area under curve. Blue line is ROC curve for diagnosing VAs originating from epicardial anterolateral portion adjacent to MA. Red line is pure opportunity line. Black dots indicate cases in epicardium group and endocardium group.

The IDT and MDI were significantly longer in the epicardium group than in the endocardium group. The values were IDT 83.8 ± 14.2 ms vs. 68.5 ± 6.5 ms (*p* < 0.01) and MDI 0.555 ± 0.089 vs. 0.463 ± 0.050 (*p* < 0.01). The ROC curve analysis showed IDT > 77 ms and MDI > 0.505, strongly indicating an epicardial origin (Figure 6).

### 3.7. Q(q)R(r) Morphology in Lead V1 Predicted the VAs Arising from the Endocardial Septal Wall Adjacent to the MA

For the VAs of endocardial septal origin, QRS morphology was often shown as Q(q)R(r) in lead V1 (53/57, 92.98%). For the VAs of epicardial anteroseptal origin, QRS morphology was often shown as rS or RS in lead V1 (34/35, 97.14%). For VAs from the epicardial anterolateral, lateral, and posterolateral walls adjacent to the MA, 92.24% (107/116) showed R or rsR’ in lead V1, and 7.76% (9/116) showed an Rs wave. The sensitivity, specificity, positive predictive value, and negative predictive value of ECG variables are shown in Table 1.

## 4. Discussion

### 4.1. Major Findings

**Figure 7:**
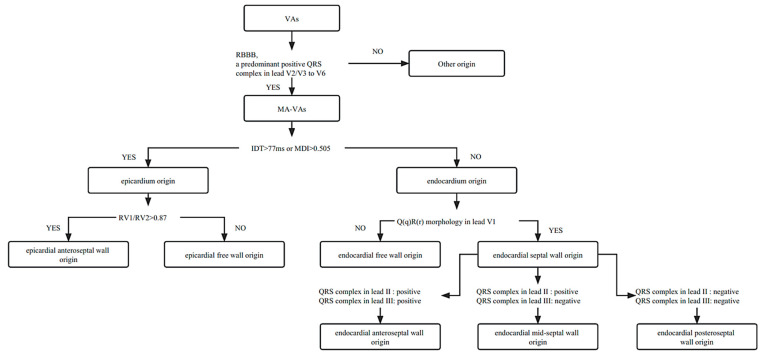
The flowchart to predict the origin of MA-VAs based on the electrocardiographic characteristics. Abbreviation: VAs, ventricular arrhythmias; MA: mitral annulus; RBBB, right bundle branch block.

The ECG characteristics of 283 patients with MA-VAs were analyzed retrospectively and the proposed algorithm to judge the origin site of MA-VAs based on the ECG variables was summarized (Figure 7). Among all MA-VAs, IDT > 77 ms or MDI > 0.505 predicted the VAs arising from the epicardium. Among all epicardial MA-VAs, the R_V1_/R_V2_ ratio > 0.87 predicted the VAs originating from the epicardial anteroseptal wall adjacent to the MA. Among all endocardial MA-VAs, Q(q)R(r) morphology in lead V1 predicted the VAs arising from the endocardial septal wall adjacent to the MA. Among all endocardial septal MA-VAs, a predominant positive wave in lead II and a predominant negative wave in lead III predicted the VAs arising from the endocardial midseptal portion adjacent to the MA.

### 4.2. ECG Characteristics of MA-VAs

MA-VAs account for 8.24% of the patients with VAs who underwent RFCA in The Second Affiliated Hospital and Yuying Children’s Hospital of Wenzhou Medical University. Previous studies have shown that some patients had ectopic atrioventricular node-like tissue on the mitral annulus, contributing to MA-VAs [18]. MA was anatomically located posterior to the left ventricle, and the direction of ventricular depolarization of MA-VAs was directly opposite to the anterior thoracic leads. It resulted in an early R-wave transition zone and predominantly positive R waves (R or Rs) in the anterior thoracic leads. Previous studies proposed an algorithm to predict MA-VAs, in which the transition zone was earlier than V2 [7,8]. However, the present study found that the transition zone was widely distributed in V1–V3 in patients with anteroseptal origin. Furthermore, S wave in lead V6 was previously considered one of the features of MA-VAs [8,19], but it was also almost impossible to observe in patients with anteroseptal origin. Above all, we believe that using an RBBB pattern and a predominant positive QRS complex in lead V2/V3 to V6 as criteria can screen out VA patients with MA origin as much as possible.

### 4.3. ECG Differences between Epicardium and Endocardium

In previous studies, pseudo-delta waves (PdWs) were measured from the QRS onset to the notching in the upstroke of the R wave and were often used to predict the epicardial VAs [5,16]. However, the present study noticed that notching could not be observed in 75.76% (100/132) of cases with VAs of endocardial origin. Therefore, PdWs could not be measured in a large number of cases. The notch was subtle in ECG, sometimes at the normal speed and normal gain of 25 mm/s and 10 mm/mV, respectively. Therefore, the authors hypothesized that there were many restrictions on the clinical application of PdWs. However, the appearance of upstroke notching indicated that VAs were more likely to originate from the epicardium. This was because of a significant acceleration in ventricular depolarization when stimulation was transferred from the epicardium to the impulse-conducting system located in the endocardium. An IDT > 77 ms and MDI > 0.505 strongly indicated epicardial origin. VAs of epicardial origin had significantly longer intervals. Purkinje fibers were located in the endocardium, and the transmission of epicardial activation took longer. Furthermore, the epicardial origin was mostly located in the free wall due to the anatomical structure limitation of the great cardiac vein. It took longer for excitement to travel from the free wall to the entire ventricle.

### 4.4. ECG Differences within Epicardium

Hayashi T et al. reported an abrupt loss of the R wave in lead V2 (R_V1_ > R_V2_), which suggests an origin anatomically opposite to lead V2 [20]. In our study, an abrupt loss of the R wave in lead V2 was observed in 16 cases of epicardial anteroseptal origin (AIV origin). It was not observed in other portions. Based on this, this study proposed that R_V1_/R_V2_ ratio can be used to predict the Vas originating from the epicardial anteroseptum.

### 4.5. ECG Differences between Endocardial Free Wall and Septum

Lead I was used to distinguish between the Vas of free wall origin and septal origin. However, Vas originated from the posterolateral portion adjacent to the MA, exciting the lateral and anterolateral walls forward and outward. They often showed a predominantly positive R wave in lead I, similar to the VAs of endocardial septal origin. In addition, Q(q)R(r) morphology appearing in lead V1 was an ideal marker, which strongly indicated the VAs from the endocardial septal wall adjacent to MA due to the septum origin excitation conducting away from lead V1 to the left ventricle.

### 4.6. ECG Difference within the Endocardial Septum

Among the endocardial septum, positive waves in inferior leads (II, III, and aVF) indicated anterior origin, while negative waves indicated posterior origin. The midseptal origin was the most common in the endocardium group, except for the anterolateral origin. From anteroseptum to posteroseptum, the R wave amplitude decreased in lead II, lead III, and aVF. The downward trends in lead II and lead III were the smallest and most significant, respectively. Therefore, 86.04% (37/43) of patients with VAs from the midseptum showed a predominant positive wave in lead II and a predominant negative wave in lead III.

### 4.7. Radiofrequency Catheter Ablation of MA-VAs

The mapping and ablation of MA-VAs were similar to that of the left manifest accessory pathway. Coronary sinus lead could be placed to provide distinct location marks, which significantly shortened the mapping time and improved the ablation’s success rate. The leading region of V wave in coronary sinus lead was often the origin region of VAs. The ECG’s prediction of the origin site before RFCA significantly sped up the RFCA process. If the ECG indicated that MA-VAs originated from the endocardial anteroseptum or midseptum, attention was paid to the effect of ablation on the His bundle to avoid the atrioventricular block. 

The possible epicardial origin of MA was considered for a few patients with ineffective endocardial ablation of the MA (a total of nine patients experienced ineffective endocardium ablation and were confirmed as epicardial origin), especially when IDT ≥ 77 ms and MDI ≥ 0.505. All of the cases in the epicardium group in this study were ablated via the coronary sinus approach. When the ablation catheter was placed in the GCV via the coronary sinus approach, coronary angiography was performed before discharge to clarify the relationship between the target and the circumflex branch of the coronary artery. If the distance between them was < 5 mm, ablation was abandoned to avoid damaging the circumflex branch of the coronary artery. It is worth noting that the impedance of the great cardiac vein was often > 250 Ω. It was difficult to accomplish effective ablation with the common temperature-controlled ablation catheter, so it was better to use a saline perfusion catheter. The saline flow rate might need to be increased to ≥60 mL/minute to overcome high impedance. The catheter insertion needs to overcome a thin lumen of GCV and several anatomical obstacles, including the Thebesian and Vieussens valves. It leads to a significantly higher complication of epicardial ablation than endocardial ablation. Coronary vein dissection caused by catheter insertion was the most common complication in the epicardium group. Due to the self-healing ability of the vascular endothelium and the effect of blood flow compression, coronary vein dissection often did not require any intervention, and no adverse events occurred in hospital and out of hospital. During ablation, more attention should be paid to changes in patients’ major complaints, physical signs, and local impedance changes to avoid serious complications.

This study involved 36 cases with low LVEF and enlarged LV. All of them suffered from cardiac dysfunction caused by VAs and recovered to normal gradually after RFCA without any complication. When patients have cardiac dysfunction, it is necessary to investigate whether there is structural heart disease and evaluate the risk of ablation.

### 4.8. Study Limitations

(i) This study was retrospective research that needs to be confirmed by prospective studies. (ii) This study was a single-center nonrandomized case–control study that needs to be verified by multicenter randomized clinical trials. (iii) This study was completed under the guidance of a three-dimensional mapping system and coronary venography. Most of the patients had not been examined using intracardiac ultrasound, which might have affected the location of effective targets.

## 5. Conclusions

The ECG characteristics of VAs from the different sites adjacent to the MA can help to judge the origin of these arrhythmias and design an ablation plan accordingly.

## Figures and Tables

**Table 1:** Sensitivity, specificity, PPV, and NPV of ECG variables to identify VAs arising from the different portions adjacent to the MA.

ECG Variables	Sensitivity (%)	Specificity (%)	PPV	NPV
R_V1_/R_V2_ ratio > 0.87 predicts the VAs originating from the epicardial anterolateral wall adjacent to the MA among all epicardial MA-VAs	62.86% (22/35)	98.06% (101/103)	91.67% (22/24)	88.60% (101/114)
IDT > 77 ms predicts the VAs arising from the epicardium among all MA-VAs	70.20% (106/151)	94.70% (125/132)	93.81% (106/113)	73.53% (125/170)
MDI > 0.505 predicts the VAs arising from the epicardium among all MA-VAs	73.51% (111/151)	82.58% (109/132)	82.84% (111/134)	73.15% (109/149)
Q(q)R(r) morphology in lead V1 predicts the VAs arising from the endocardial septal wall adjacent to the MA among all endocardial MA-VAs	92.98% (53/57)	100% (75/75)	100% (53/53)	94.94% (75/79)
A predominant positive wave in lead II and a predominant negative wave in lead III predict the VAs arising from the endocardial midseptal portion adjacent to the MA among all endocardial septal MA-VAs	86.04% (37/43)	100% (14/14)	100% (37/37)	70.00% (14/20)

MA, mitral annulus; IDT, intrinsicoid deflection time; MDI, maximum deflection index; PPV, positive predictive value; NPV, negative predictive value.

## Data Availability

Data are available on reasonable request to the corresponding author.

## Supplementary Materials

The following supporting information can be downloaded at: https://www.mdpi.com/article/10.3390/jcdd10080334/s1. Table S1: Comparison of patient characteristics between the two groups; Table S2: Comparison of electrophysiological study and radiofrequency catheter ablation between two groups; Table S3: Complications of radiofrequency catheter ablation in two groups; Table S4: The QRS morphology of VAs originating from the area adjacent to mitral annulus in two groups.
