# Supplementary material for: Electrocardiographic Characteristics of Ventricular Arrhythmias Originating from Different Areas Adjacent to the Mitral Annulus
Source: J Cardiovasc Dev Dis. 2023 Aug 3;10(8):334. doi: 10.3390/jcdd10080334 (PMC10455119; doi:10.3390/jcdd10080334)
Supplement: Supplementary file 1 [file jcdd-10-00334-s001.zip › jcdd-2487016-supplementary.pdf]

Supplementary Table S1. Comparison of patient characteristics between the two groups.

	Group		
	**Epicardium group** (*n* = 151)	**Endocardium group** (*n* = 132)	***P* Value**
Sex (Male %)	93(61.6%)	80(60.6%)	>0.05
Age (year)	56.8±15.6	59.8±15.0	>0.05
Total number of PVCs (times/24h)	27566±10143	26547±9028	>0.05
Disease duration (year)	2.7±1.9	2.9±1.9	>0.05
Hypertension	24(15.9%)	19(14.4%)	>0.05
Diabetes	16(10.6%)	15(11.4%)	>0.05
Left ventricular hypertrophy	21(13.9%)	15(11.4%)	>0.05
NSVT/SVT	34(22.5%)	26(18.2%)	>0.05
PVCs	117(77.5%)	108(81.8%)	>0.05
LVEDd(mm)	48.9±5.0	48.0±4.8	>0.05
LVEF (%)	65.7±6.7	66.6±5.3	>0.05

PVC indicates premature ventricular contraction; LVEF, left ventricular ejection fraction; LVEDd, left ventricular end-diastolic dimension; NSVT, non-sustained ventricular tachycardia; SVT, sustained ventricular tachycardia.

Supplementary Table S2. Comparison of electrophysiological study and radiofrequency catheter ablation between two groups (case, %).

	Group		
	**Epicardium group** (*n* = 151)	**Endocardium group** (*n* = 132)	***P* Value**
V-QRS, ms	−34.7±4.9	−33.2±5.2	>0.05
Ventricular capture	133(88.1%)	120(90.9%)	>0.05
Distance between target site and LAD< 5 mm	7(4.6%)	0(0)	<0.05
Number of similar lead counts	11.5±0.9	11.2±0.9	>0.05
fluoroscopy time, min	9.8±2.5	5.3±1.7	<0.05
Procedure time, min	68.0±9.1	57.4±7.2	<0.05
Swartz sheath support	93(61.6%)	3(2.3)	<0.05
Acute success	129(85.4%)	126(95.5%)	<0.05
Presystolic long-duration multicomponent fractionated potential in bipolar mapping*	46(35.7%)	13(10.3%)	<0.05
Large amplitude potential in bipolar mapping	4(3.5%)	3(2.4%)	>0.05
QS wave in unipolar mapping	129(100.0%)	126(100.0%)	>0.05
Notch in the downstroke of the QS wave in unipolar mapping	49(37.98%)	38(30.2%)	>0.05
Reaching the target	144(95.4%)	132(100.0%)	<0.05
Ablation time, s	164.3±42.2	206.9±54.5	<0.05
Energy delivered times	2.6±1.2	2.4±0.9	>0.05

CS indicates coronary sinus; PGCV, proximal great coronary vein; MGCV, middle great coronary vein; DGCV1, distal great coronary vein (The epicardium of the anterior wall of mitral annulus); DGCV2, distal great coronary vein (The orifice of anterior interventricular vein); AIV, Anterior Interventricular Vein; Summit-CV, summit communicating vein; and LAD, left anterior descending coronary artery. * Presystolic long-duration multicomponent fractionated potential is a fractionated potential with multiple (more than 3), fast deflections, whose duration occupied more than 50% ventricular electrogram duration, with high-amplitude or low-amplitude potential.

Supplementary Table S3. Complications of radiofrequency catheter ablation in two groups.

Complications	Epicardium group (n = 151)	Endocardium group (n=132)
Coronary vein dissection	8(5.29%)	0
Coronary vein rupture	2(1.32%) a, b	0
Acute pericardial effusion	2(1.32%) a, b	0
Coronary artery injury	3(1.99%) c, d, e	0
Coronary artery spasm	2(1.32%) c, d	0
Delayed pericardial effusion	1(0.66%) e	0
Acute occlusion of the first diagonal branch	2(1.32%)	0
Steam pops	0	4(3.03%)
Death	0	0
Sum	16(9.93%)	4(3.03%)

a, b, c, d and e represent the same patient.

Supplementary Table S4. The QRS morphology of VAs originating from the area adjacent to mitral annulus in two groups.

Group	Cases	I	II、III、aVF	aVL	aVR	V1	V2, V3	V5, V6
**Epicardium group**	151							
Anterolateral	103	3Rs, 58rs, 42qs	103R	103QS	103QS	94R, 9Rs	48R, 55Rs	67R, 36Rs
Lateral	5	4rs, 1R	1R, 1Rs, 2rs, 1QS	5R	2QS, 3R	5R	4R, 1Rs	2R, 3Rs
Posterolateral	8	8R	8QS	3R, 5Rs	6R, 2qr	8R	8R	3R, 5Rs
Anteroseptal	35	24rs, 2qr, 9qs	35R	35R	35QS	21rS, 13RS, 1R	19rS, 16RS	35R
**Endocardium group**	132							
Anterolateral	66	61rS, 5qs	66R	66QS	66QS	57R, 9Rs	22R, 34Rs	49Rs, 17R
Lateral	3	1R,2rS	2R, 1RS	2QS, 1r	3Qr	3R	2R, 1Rs	1R, 2Rs
Posterolateral	6	5R,1rS	1Rs, 5rs/QS	5R, 1Rs	5QS, 1qr	1r′sR, 5R	5R, 1Rs	2R, 4Rs
Posteroseptal	10	10R	10rS	10R	10Qr	5qR, 3qr, 2R	8R, 2Rs	4R, 6Rs
Midseptal	43	43R	9Rs, 34rs/QS	43R	43Qr/QS	27qR, 14qr, 2R	35R, 8Rs	28R, 15Rs
Anteroseptal	4	4R	1Rs or 3R	4R	4QS	3qR, 1qr	3R, 1rsR′	4R

The number before the letter represents the number of cases with such wave. Capital letters (Q, R, S) represented waves of relatively high amplitude (>0.5 mV), while lowercase letters (q, r, s) represented waves of relatively low amplitude (<0.5 mV).
